# 6-Meth­oxy-2-methyl-1-*m*-tolyl-1*H*-benzimidazole hemihydrate

**DOI:** 10.1107/S1600536811031060

**Published:** 2011-08-06

**Authors:** Xu-Bin Fang, Lei Fang, Xu-Ying Liu

**Affiliations:** a Department of Chemistry and Chemical Engineering, Southeast University, Nanjing, People’s Republic of China

## Abstract

The title compound, C_16_H_16_N_2_O·0.5H_2_O, is a substituted 1-phenyl­benzimidazole, which belongs to the class of ATP-site inhibitors of the platelet-derived growth-factor receptor. In the crystal, the components are linked by an O—H⋯N hydrogen bond.

## Related literature

For related structures, see: Zhong (2004 ▶). For medicinal background, see: Palmer (1998 ▶).
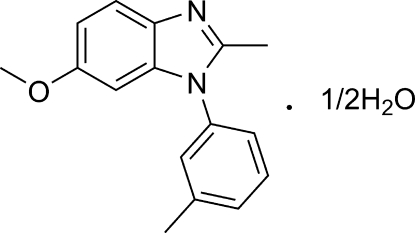


## Experimental

### 

#### Crystal data

C_16_H_16_N_2_O·0.5H_2_O
*M* *_r_* = 261.32
Orthorhombic, 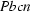

*a* = 16.0752 (16) Å
*b* = 13.9140 (14) Å
*c* = 12.6450 (13) Å
*V* = 2828.3 (5) Å^3^
*Z* = 8
Mo *K*α radiation
μ = 0.08 mm^−1^
*T* = 293 K
0.25 × 0.23 × 0.21 mm

#### Data collection

Bruker APEX CCD diffractometer
Absorption correction: multi-scan (*SADABS*; Sheldrick, 1996 ▶) *T*_min_ = 0.980, *T*_max_ = 0.983
13133 measured reflections
2720 independent reflections
2018 reflections with *I* > 2σ(*I*)
*R*_int_ = 0.029

#### Refinement

*R*[*F* ^2^ > 2σ(*F* ^2^)] = 0.081
*wR*(*F* ^2^) = 0.155
*S* = 1.03
2720 reflections
178 parameters
H-atom parameters constrained
Δρ_max_ = 0.69 e Å^−3^
Δρ_min_ = −0.20 e Å^−3^

### 

Data collection: *SMART* (Bruker, 2000 ▶); cell refinement: *SAINT* (Bruker, 2000 ▶); data reduction: *SAINT*; program(s) used to solve structure: *SHELXS97* (Sheldrick, 2008 ▶); program(s) used to refine structure: *SHELXL97* (Sheldrick, 2008 ▶); molecular graphics: *SHELXTL* (Sheldrick, 2008 ▶); software used to prepare material for publication: *SHELXTL*.

## Supplementary Material

Crystal structure: contains datablock(s) I, global. DOI: 10.1107/S1600536811031060/aa2011sup1.cif

Structure factors: contains datablock(s) I. DOI: 10.1107/S1600536811031060/aa2011Isup2.hkl

Supplementary material file. DOI: 10.1107/S1600536811031060/aa2011Isup3.cml

Additional supplementary materials:  crystallographic information; 3D view; checkCIF report

## Figures and Tables

**Table 1:** Hydrogen-bond geometry (Å, °)

*D*—H⋯*A*	*D*—H	H⋯*A*	*D*⋯*A*	*D*—H⋯*A*
O1*W*—H1*W*⋯N2	0.85	2.08	2.911 (3)	166

**Fig. 1.**
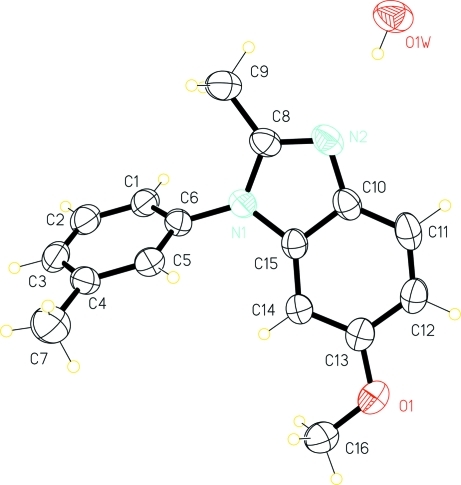
The asymmetric unit of the title compound. Displacement ellipsoids are drawn at the 30% probability level.

**Fig. 2.**
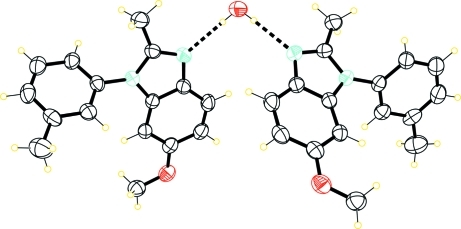
The dimeric structure of the title compound is built via O—H···N hydrogen bonding between water molecule and N atoms of imidazole rings.

## supplementary crystallographic information

### Comment

1-Phenylbenzimidazoles are shown to be a new class of ATP-site inhibitors of the platelet derived growth factor receptor (PDGFR), with clear evidence of the relationship between their molecular features and their inhibitory activity (Palmer, 1998). However, few structure-activity relationship studies involving 1-phenylbenzimidazoles have been published and the QSAR models reported were not completely satisfactory (Zhong, 2004). The synthesis of these compounds is relatively uncomplicated, many methods have been proposed in the past years. We have successfully synthesized the title compound as a key analogue of 1-phenylbenzimidazole.

### Experimental

*N*-(2-amino-5-methoxyphenyl)-*N*-*m*-tolylacetamide (1 g, 3.70 mmol) was dissolved in 40 ml of 18% hydrochloric acid, and the solution was cooled to 0°C. A solution of NaNO_2_ (0.28 g, 4.07 mmol) in 1 ml of water was added under stirring, and the mixture was stirred for 10 min. Copper powder (1 g) was then added, and the mixture was stirred for 30 min at room temperature. The reaction solution was heated to 70°C and stirred for 3 h. The mixture was extracted with ethyl acetate (2×50 ml), and the extract was washed with a 3% aqueous solution of NaHCO_3_ and water, respectively. The organic phase was dried over Na_2_SO_4_ and evaporated. The residue was purified by column chromatography. Yield 0.46 g (49%).

### Refinement

The H atoms were positioned geometrically and allowed to ride on their parent atoms, with C—H = 0.93–0.96 Å, and *U*_iso_ =1.2 or 1.5U_eq_(parent atom). The maximum residual electron density, 0.685 e/Å^3^, is located at 0.7013, 0.6556, 0.0873 (1.319 Å from C2 atom). This can be explained by a little disorder of the C7 methyl group.

### Crystal data

**Table:**

C_16_H_16_N_2_O·0.5H_2_O	*F*(000) = 1112
*M**_r_* = 261.32	*D*_x_ = 1.227 Mg m^−^^3^
Orthorhombic, *P**b**c**n*	Mo *K*α radiation, λ = 0.71073 Å
Hall symbol: -P 2n 2ab	Cell parameters from 2720 reflections
*a* = 16.0752 (16) Å	θ = 2.4–28.0°
*b* = 13.9140 (14) Å	µ = 0.08 mm^−^^1^
*c* = 12.6450 (13) Å	*T* = 293 K
*V* = 2828.3 (5) Å^3^	Plate-like, colourless
*Z* = 8	0.25 × 0.23 × 0.21 mm

### Data collection

**Table:**

Bruker APEX CCD diffractometer	2720 independent reflections
Radiation source: fine-focus sealed tube	2018 reflections with *I* > 2σ(*I*)
graphite	*R*_int_ = 0.029
φ and ω scans	θ_max_ = 26.0°, θ_min_ = 2.5°
Absorption correction: multi-scan (*SADABS*; Sheldrick, 1996)	*h* = −17→19
*T*_min_ = 0.980, *T*_max_ = 0.983	*k* = −17→17
13133 measured reflections	*l* = −15→14

### Refinement

**Table:**

Refinement on *F*^2^	Secondary atom site location: difference Fourier map
Least-squares matrix: full	Hydrogen site location: inferred from neighbouring sites
*R*[*F*^2^ > 2σ(*F*^2^)] = 0.081	H-atom parameters constrained
*wR*(*F*^2^) = 0.155	*w* = 1/[σ^2^(*F*_o_^2^) + (0.0196*P*)^2^ + 3.0292*P*] where *P* = (*F*_o_^2^ + 2*F*_c_^2^)/3
*S* = 1.03	(Δ/σ)_max_ < 0.001
2720 reflections	Δρ_max_ = 0.69 e Å^−^^3^
178 parameters	Δρ_min_ = −0.20 e Å^−^^3^
0 restraints	Extinction correction: *SHELXL97* (Sheldrick, 2008), Fc^*^=kFc[1+0.001xFc^2^λ^3^/sin(2θ)]^-1/4^
Primary atom site location: structure-invariant direct methods	Extinction coefficient: 0.0037 (5)

### Special details

**Table:**

Geometry. All s.u.'s (except the s.u. in the dihedral angle between two l.s. planes) are estimated using the full covariance matrix. The cell s.u.'s are taken into account individually in the estimation of s.u.'s in distances, angles and torsion angles; correlations between s.u.'s in cell parameters are only used when they are defined by crystal symmetry. An approximate (isotropic) treatment of cell s.u.'s is used for estimating s.u.'s involving l.s. planes.
Refinement. Refinement of *F*^2^ against ALL reflections. The weighted *R*-factor *wR* and goodness of fit *S* are based on *F*^2^, conventional *R*-factors *R* are based on *F*, with *F* set to zero for negative *F*^2^. The threshold expression of *F*^2^ > σ(*F*^2^) is used only for calculating *R*-factors(gt) *etc*. and is not relevant to the choice of reflections for refinement. *R*-factors based on *F*^2^ are statistically about twice as large as those based on *F*, and *R*- factors based on ALL data will be even larger.

### Fractional atomic coordinates and isotropic or equivalent isotropic displacement parameters (Å^2^)

**Table:**

	*x*	*y*	*z*	*U*_iso_*/*U*_eq_	
C1	0.6204 (2)	0.6782 (2)	0.2566 (2)	0.0725 (8)	
H1A	0.5851	0.6253	0.2539	0.087*	
C2	0.6771 (2)	0.6930 (3)	0.1781 (3)	0.0851 (10)	
H2A	0.6809	0.6497	0.1223	0.102*	
C3	0.7285 (2)	0.7711 (3)	0.1814 (3)	0.0863 (11)	
H3A	0.7671	0.7800	0.1274	0.104*	
C4	0.72472 (19)	0.8370 (2)	0.2623 (3)	0.0789 (10)	
C5	0.66713 (18)	0.8218 (2)	0.3437 (3)	0.0699 (8)	
H5A	0.6636	0.8650	0.3996	0.084*	
C6	0.61540 (17)	0.7422 (2)	0.3404 (2)	0.0624 (7)	
C8	0.5560 (2)	0.6505 (2)	0.4939 (2)	0.0681 (8)	
C9	0.6135 (2)	0.5667 (2)	0.4845 (3)	0.0848 (10)	
H9A	0.6020	0.5215	0.5401	0.127*	
H9B	0.6700	0.5884	0.4903	0.127*	
H9C	0.6055	0.5361	0.4172	0.127*	
C10	0.4610 (2)	0.7482 (2)	0.5473 (2)	0.0681 (8)	
C11	0.3959 (2)	0.7953 (3)	0.5984 (3)	0.0819 (10)	
H11A	0.3717	0.7687	0.6585	0.098*	
C12	0.3683 (2)	0.8807 (3)	0.5596 (3)	0.0756 (9)	
H12A	0.3245	0.9120	0.5932	0.091*	
C13	0.40456 (18)	0.9223 (2)	0.4699 (2)	0.0677 (8)	
C14	0.46999 (17)	0.8783 (2)	0.4176 (2)	0.0611 (7)	
H14A	0.4946	0.9056	0.3583	0.073*	
C15	0.49675 (18)	0.7910 (2)	0.4588 (2)	0.0592 (7)	
C16	0.4029 (2)	1.0538 (3)	0.3480 (3)	0.0863 (10)	
H16A	0.3731	1.1123	0.3341	0.129*	
H16B	0.3979	1.0116	0.2883	0.129*	
H16C	0.4605	1.0683	0.3601	0.129*	
N1	0.55804 (15)	0.72736 (17)	0.42521 (18)	0.0628 (6)	
N2	0.49971 (19)	0.66035 (18)	0.5677 (2)	0.0764 (7)	
O1	0.36911 (13)	1.00859 (17)	0.43877 (19)	0.0811 (7)	
O1W	0.5000	0.5327 (2)	0.7500	0.0961 (11)	
H1W	0.4912	0.5668	0.6953	0.144*	
C7	0.7786 (3)	0.9187 (3)	0.2623 (4)	0.1196 (16)	
H7A	0.8138	0.9166	0.2011	0.179*	
H7B	0.8123	0.9180	0.3250	0.179*	
H7C	0.7459	0.9764	0.2609	0.179*	

### Atomic displacement parameters (Å^2^)

**Table:**

	*U*^11^	*U*^22^	*U*^33^	*U*^12^	*U*^13^	*U*^23^
C1	0.077 (2)	0.076 (2)	0.0645 (18)	0.0169 (17)	0.0040 (16)	0.0042 (16)
C2	0.092 (2)	0.093 (3)	0.070 (2)	0.029 (2)	0.0057 (19)	0.0060 (19)
C3	0.081 (2)	0.104 (3)	0.074 (2)	0.038 (2)	0.0189 (18)	0.019 (2)
C4	0.0568 (18)	0.074 (2)	0.106 (3)	0.0035 (16)	0.0035 (18)	0.033 (2)
C5	0.0636 (18)	0.0701 (19)	0.076 (2)	0.0092 (16)	0.0030 (15)	0.0106 (16)
C6	0.0602 (17)	0.0685 (18)	0.0584 (16)	0.0054 (15)	0.0027 (13)	0.0111 (14)
C8	0.083 (2)	0.0562 (17)	0.0649 (18)	−0.0107 (16)	0.0003 (16)	0.0009 (14)
C9	0.110 (3)	0.066 (2)	0.079 (2)	0.0055 (19)	−0.004 (2)	0.0036 (17)
C10	0.077 (2)	0.0635 (18)	0.0634 (17)	−0.0187 (16)	0.0101 (15)	−0.0055 (14)
C11	0.083 (2)	0.089 (2)	0.074 (2)	−0.025 (2)	0.0264 (18)	−0.0117 (18)
C12	0.0671 (19)	0.079 (2)	0.081 (2)	−0.0068 (17)	0.0137 (17)	−0.0201 (18)
C13	0.0566 (17)	0.075 (2)	0.0714 (19)	−0.0066 (15)	−0.0019 (15)	−0.0158 (16)
C14	0.0597 (16)	0.0658 (17)	0.0579 (16)	−0.0049 (14)	0.0025 (13)	−0.0022 (14)
C15	0.0563 (15)	0.0632 (17)	0.0582 (15)	−0.0087 (14)	0.0045 (13)	−0.0069 (13)
C16	0.090 (2)	0.081 (2)	0.088 (2)	0.011 (2)	−0.005 (2)	0.0008 (19)
N1	0.0676 (15)	0.0610 (14)	0.0599 (13)	−0.0022 (12)	0.0055 (12)	0.0042 (11)
N2	0.0991 (19)	0.0625 (15)	0.0677 (16)	−0.0165 (15)	0.0114 (15)	0.0024 (13)
O1	0.0725 (13)	0.0838 (15)	0.0870 (16)	0.0143 (12)	−0.0013 (12)	−0.0094 (13)
O1W	0.144 (3)	0.0619 (19)	0.082 (2)	0.000	0.002 (2)	0.000
C7	0.100 (3)	0.097 (3)	0.163 (4)	−0.008 (2)	0.008 (3)	0.013 (3)

### Geometric parameters (Å, °)

**Table:**

C1—C2	1.363 (5)	C10—C11	1.394 (4)
C1—C6	1.387 (4)	C10—N2	1.395 (4)
C1—H1A	0.9300	C11—C12	1.359 (5)
C2—C3	1.366 (5)	C11—H11A	0.9300
C2—H2A	0.9300	C12—C13	1.401 (4)
C3—C4	1.375 (5)	C12—H12A	0.9300
C3—H3A	0.9300	C13—C14	1.385 (4)
C4—C5	1.400 (4)	C13—O1	1.386 (4)
C4—C7	1.429 (5)	C14—C15	1.389 (4)
C5—C6	1.385 (4)	C14—H14A	0.9300
C5—H5A	0.9300	C15—N1	1.391 (4)
C6—N1	1.429 (3)	C16—O1	1.417 (4)
C8—N2	1.307 (4)	C16—H16A	0.9600
C8—N1	1.378 (4)	C16—H16B	0.9600
C8—C9	1.492 (4)	C16—H16C	0.9600
C9—H9A	0.9600	O1W—H1W	0.8500
C9—H9B	0.9600	C7—H7A	0.9600
C9—H9C	0.9600	C7—H7B	0.9600
C10—C15	1.392 (4)	C7—H7C	0.9600
			
C2—C1—C6	119.9 (3)	C12—C11—H11A	120.4
C2—C1—H1A	120.1	C10—C11—H11A	120.4
C6—C1—H1A	120.1	C11—C12—C13	121.2 (3)
C1—C2—C3	120.2 (4)	C11—C12—H12A	119.4
C1—C2—H2A	119.9	C13—C12—H12A	119.4
C3—C2—H2A	119.9	C14—C13—O1	124.1 (3)
C2—C3—C4	121.7 (3)	C14—C13—C12	121.3 (3)
C2—C3—H3A	119.1	O1—C13—C12	114.7 (3)
C4—C3—H3A	119.1	C13—C14—C15	116.3 (3)
C3—C4—C5	118.4 (3)	C13—C14—H14A	121.9
C3—C4—C7	120.2 (4)	C15—C14—H14A	121.9
C5—C4—C7	121.4 (4)	C14—C15—N1	131.4 (3)
C6—C5—C4	119.7 (3)	C14—C15—C10	123.2 (3)
C6—C5—H5A	120.1	N1—C15—C10	105.4 (3)
C4—C5—H5A	120.1	O1—C16—H16A	109.5
C5—C6—C1	120.1 (3)	O1—C16—H16B	109.5
C5—C6—N1	118.8 (3)	H16A—C16—H16B	109.5
C1—C6—N1	121.2 (3)	O1—C16—H16C	109.5
N2—C8—N1	112.6 (3)	H16A—C16—H16C	109.5
N2—C8—C9	124.6 (3)	H16B—C16—H16C	109.5
N1—C8—C9	122.8 (3)	C8—N1—C15	106.6 (2)
C8—C9—H9A	109.5	C8—N1—C6	126.9 (3)
C8—C9—H9B	109.5	C15—N1—C6	126.4 (2)
H9A—C9—H9B	109.5	C8—N2—C10	105.7 (3)
C8—C9—H9C	109.5	C13—O1—C16	117.2 (2)
H9A—C9—H9C	109.5	C4—C7—H7A	109.5
H9B—C9—H9C	109.5	C4—C7—H7B	109.5
C15—C10—C11	118.8 (3)	H7A—C7—H7B	109.5
C15—C10—N2	109.8 (3)	C4—C7—H7C	109.5
C11—C10—N2	131.4 (3)	H7A—C7—H7C	109.5
C12—C11—C10	119.2 (3)	H7B—C7—H7C	109.5

### Hydrogen-bond geometry (Å, °)

**Table:**

*D*—H···*A*	*D*—H	H···*A*	*D*···*A*	*D*—H···*A*
O1W—H1W···N2	0.85	2.08	2.911 (3)	166.
